# Influence of *Potamogeton crispus* harvesting on phosphorus composition of Lake Yimeng

**DOI:** 10.1038/s41598-022-22484-7

**Published:** 2022-10-21

**Authors:** Lizhi Wang, Xiyuan Wu, Hongli Song, Juan An, Bin Dong, Yuanzhi Wu, Yun Wang, Bao Li, Qianjin Liu, Wanni Yu

**Affiliations:** Shandong Provincial Key Laboratory of Water and Soil Conservation and Environmental Protection, College of Resources and Environment, Linyi University, Linyi, 276005 China

**Keywords:** Element cycles, Environmental chemistry

## Abstract

Harvesting is an important method used to control the overproduction of *Potamogeton crispus* in lakes. A three-year comparative field study was performed in a eutrophic lake (harvested area) and its connected lake (non-harvested area) to determine the effects of harvesting on the phosphorus (P) composition and environmental factors in the water and sediment. Results revealed that harvesting significantly reduced the dissolved total P and dissolved organic P (DOP) and increased the alkaline phosphatase activity and particulate P (PP) in the water. No significant differences were detected in the water total P (TP), soluble reactive P, chlorophyll-a, pH, and dissolved oxygen between the harvested and non-harvested areas. Sediment TP and organic P (OP) were significantly reduced in the harvested area. Harvesting changed the P composition in the water. In the non-harvested area, P was mainly formed by DOP (40%) in the water body, while in the harvested area, PP was the main water component (47%). Harvesting increased the proportion of inorganic P (IP) in the sediment and decreased the proportion of OP. In the water, the IP to TP ratio in the non-harvested and harvested areas were 58.26% and 63.51%, respectively. Our results showed that harvesting changed the P composition in the water and sediment. In the harvesting of submerged vegetation, our results can serve as a reference for the management of vegetation-rich lakes.

## Introduction

Submerged macrophytes as primary producers play important roles in maintaining the structure, function, and biodiversity of lake ecosystems^1^. Submerged macrophytes secrete allelochemicals, inhibit the propagation of algae, and absorb nutrients in the overlying water and sediment, which has important ecological value in controlling lake eutrophication^2^. The restoration, reconstruction, and transformation of aquatic plants have become important methods in the ecological regulation and endogenous pollution load control of shallow lakes^3^. However, when a large number of submerged macrophytes decompose, the residue still exists in the water and decomposes as well, which releases nitrogen (N), phosphorus (P), and other raw elements into the overlying water, thereby leading to secondary pollution^4^. Harvesting is an important method used for the overgrowth of submerged macrophytes in lakes. Harvesting submerged macrophytes is conducive not only to the recovery and growth of plants, which increases biodiversity, but also to improving the stability of the community to continuously purify the water. It can also reduce the nutrient load in a lake by harvesting plants or transferring nutrients from the lake. Additionally, harvesting can prevent the negative effects of excessive submerged macrophyte growth^5^. For submerged macrophytes whose biomass is mainly concentrated in the upper layer or surface of the water body, harvesting can alleviate excessive biomass concentration^6^. Therefore, it is of great importance to study the effects of harvesting on aquatic environments.

Harvesting directly affects the N and P cycles in the water. Currently, due to excess N, P, and other nutrients, submerged macrophytes overgrow and bloom in many lakes^7^. As the seasons change, the decomposition of many submerged macrophyte residues causes serious secondary pollution, which poses a great threat to the safety of aquatic ecosystems^8^. Decomposition and death are necessary stages in the life history of submerged macrophytes. In the field of lake ecology, the investigation of these processes is currently a popular research area^9^. In the process of natural succession and seasonal change, if aquatic plants are not harvested, then their litter will disperse into the water and sediment, and their decomposition products (organic matter, N, P, and other nutrients) will participate in the biogeochemical cycles of lake nutrients again^10^. Therefore, the decomposition of aquatic plants is the key link between material cycling and energy flow. This process will reduce the transparency of the water body and increase the contents and ratios of organic matter, N, P, and other pollutants in the water body and sediment, which will thereby result in secondary pollution^11^.

The P status in lakes is an important factor of submerged macrophytes that affects eutrophication^12^. The main forms of phosphorus in water bodies are dissolved organic P (DOP) and particulate P (PP). Soluble reactive P (SRP), can be directly utilized by phytoplankton. Although DOP and PP cannot be directly utilized by phytoplankton, they can be converted into SRP under certain conditions, which can then be absorbed and utilized by phytoplankton^13^. The relationship between eutrophication and limiting nutrients (mostly P) differs between lakes with and without submerged vegetation. Therefore, it is of great importance to explore the effects of harvesting on the P cycle in the water body to assist the management of vegetative lakes^14^.

*Potamogeton crispus* is a submerged macrophyte widely distributed throughout China that is often used in the ecological restoration of eutrophic lakes^15^. Most accomplishments in efforts to restore eutrophic lakes have been attributed to the success of aquatic macrophytic vegetation^16^. *P. crispus* germinates in autumn and grows during the winter; large reproductive growth occurs in April and May, and plants finally decompose and die in the summer. It has a rapid growth rate, can tolerate high nutrient-rich environments, and grows well in contaminated water bodies^17^.

The aim of this work was to determine how harvesting submerged macrophytes (*P. crispus*) affects the P composition and environmental factors in the water. We compared the P in the water and sediment, chlorophyll-a (Chl-a), and environmental factors between the harvested and non-harvested areas of the lake for three years. The relationship between Chl-a and P in the water and environmental factors are also discussed.

## Materials and methods

### Study area and sampling

**Figure 1:**
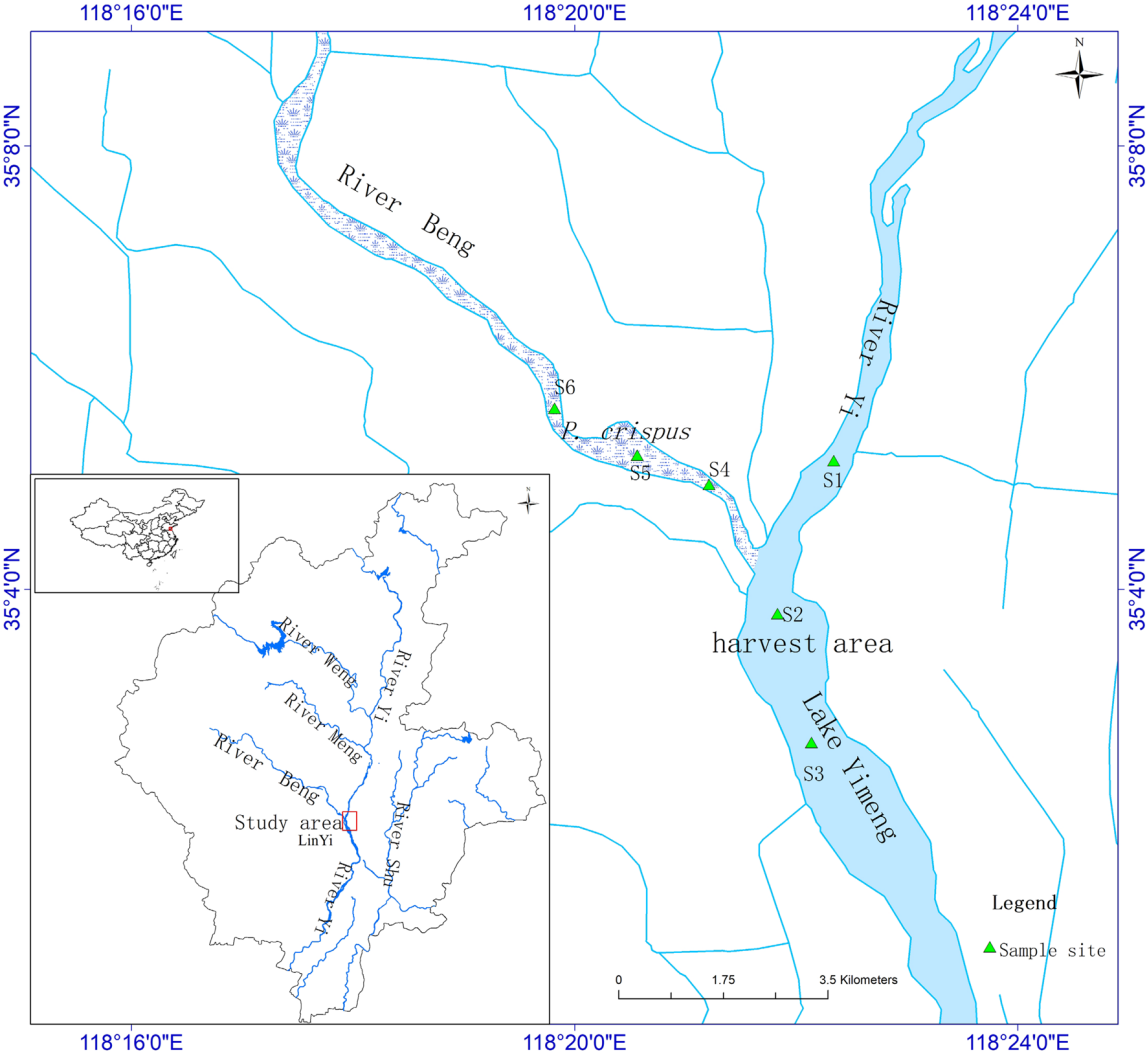
Location of studied area and its sampling sites. Map (https:// www.esri. com/) generated using ArcMap version 10.8.

Lake Yimeng was formed in 1997 when a rubber dam (1135 m) was built across the Yi River, capturing 12 million m^3^ of water. In recent decades, the lake has exhibited dense canopy-forming populations of *P. crispus*, which covers nearly 90% of the lake during spring and summer^8^. Different types of management strategies have been implemented to reduce *P. crispus* overgrowth, including harvesting during the summer. Almost all of the *P. crispus* in this area was harvested by a weed-cutting launch. The *P. crispus* that was 15 cm and higher above the sediment was harvested. According to estimates of *P. crispus* phosphorus content, about 5600 kg of the phosphorus in the lake was removed in 2017. However, in the Beng River part of the lake, *P. crispus* was not harvested. Therefore, the harvested and non-harvested areas of the lake were selected to study the effects of harvesting on P composition in the water (Fig. 1).

For several years, *P. crispus* has bloomed in Lake Yimeng, which is a eutrophic lake. The Beng River part of the lake (non-harvested *P. crispus* area) is connected to the Yi River part of the lake (35°2′40″–35°7′11″N; 118°22′11″–118°23′12″E). Three corresponding sampling sites were selected in the non-harvested and harvested areas (Fig. 1). Beng River was completely covered by *P. crispus* in its growing season with a mean biomass of 1025 g·m^−2^. Although the Beng River is connected to the Yi River, there is little water exchange. During the rainy season from July to August, water flows from the Beng River to the Yi River. The Secchi disc depth varies between 20 and 60 cm in the Yi River and between 50 and 145 cm in the Beng River.

Mixed water samples from the upper layer of each sampling site (0, 0.5, and 1 m) were collected using a Grasp sampler (Grasp BC-2300, Beijing, China) every month. Afterwards, water samples were stored in a refrigerator at 4 °C for further analysis. For total P (TP) analysis, water samples were autoclaved at 121 °C for 30 min after the addition of K_2_S_2_O_8_. The samples were measured using the molybdenum blue spectrophotometric method^18^. A continuous flow analyzer (Flowsys III, Systea Company, Italy) was used to determine the P concentration. The same method was used for the soluble reactive P (SRP) determination, except the water samples were filtered through a 0.45 μm cellulose acetate membrane and not autoclaved. Dissolved total P (DTP) was measured using the same method as TP, except the water samples were filtered through a 0.45 μm cellulose acetate membrane. The difference between the TP and DTP was defined as the sum of the particulate P (PP) fraction. The difference between the DTP and SRP was defined as the dissolved organic P (DOP) fraction. The detection limit for the SRP and DTP concentrations in the overlying water was 1 μg L^−1^. All materials used for the analyses were purchased from the Shanghai N&D Co., Ltd. (Shanghai, China). For all samples, triplicates were analyzed and the data are expressed as the mean. The P concentration was determined according to previously described methods of Goulden and Brooksbank^19^.

Two liters were collected for measuring the physical and chemical indices, such as Chl-a,APA content. Sediment samples from the top 10 cm at each sampling site were collected at the same time when mixed water samples were collected. Sediment samples were collected using a self-made hand-driven polyethylene corer (patent No. ZL201420135437.1), immediately transported to the lab in sealed plastic bags placed in iceboxes, and then freeze-dried and sieved (< 2 mm). All samples were stored at 0–4 °C for further analysis.

Sediment P fractionation of the TP, inorganic P (IP), and organic P (OP) was conducted based on part of the Standards Measurements and Testing (SMT) protocol, which consists of three extraction procedures applied to 0.2 g aliquots of sediment samples. The SMT protocol is a common approach used for studying the P fractions of lake sediment^20^. Extractions (16 h) used 1 mol·L^−1^ HCl and were performed to remove the IP. The residues from the extractions were placed in a porcelain crucible and calcined in a furnace for 3 h at 450 °C. The residues were extracted (16 h) again using 1 mol·L^−1^ HCl to remove P associated with the organic matter of the sediment (OP). To obtain the TP, a simple extraction (16 h) using 3.5 mol L^−1^ HCl was performed after the calcination of a separate sample for 3 h at 450 °C. For all cases, phosphate was determined in the extracts via a spectrophotometric procedure. Measurements were conducted at 886.0 nm.

The extracellular alkaline phosphatase activity (APA) in the water samples was determined following the previously described methods of Gage and Gorham^21^. Water samples were filtered through 0.45 μm Millipore filters to determine the Chl-a content using an extraction of 90% acetone^22^.

The pH value was measured using a PHSJ-4A (Lei-ci, Shanghai, China) and the dissolved oxygen (DO) concentration was measured using a YSI 5750(USA) before sampling in each site. The final values were the average of the measurements taken 10 cm below the surface, from the middle of the lake, and about 10–15 cm above the bottom. The *P. crispus* within a 1 square meter section of the lake was collected. The water on the stem and leaf surfaces of *P. crispus* was dried using absorbent paper, and then the *P crispus* was weighed.

## Statistical analysis

Correlation analyses were performed using SPSS v25.0 (IBM Corp., Chicago, IL, USA). Differences were considered statistically significant at *P* < 0.05 and *P* < 0.01. Tukey’s post-hoc test after two-way ANOVA was used to determine the differences between the harvested and non-harvested areas of the water and sediment indices of different sections. The relationship between the water chemical and physical parameters of the two areas were assessed using Pearson correlation analyses. The results are presented as the average of three replicates. The default Z-scores in SPSS were adopted for data standardization. ArcMap 10.8 was used to draw the maps (https://www.esri.com/). Principal component analysis (PCA) was used for data dimensions, and the extraction method was used for principal components.

## Results

### Monthly variations in water P composition and physical parameters

During the rainy season, some river water flows into the harvested area from the non-harvested area, which affects the P concentration of S2 and S3. Therefore, the S1, S2 and S3 in the harvested area were compared with those in the non-harvested area.

**Figure 2:**
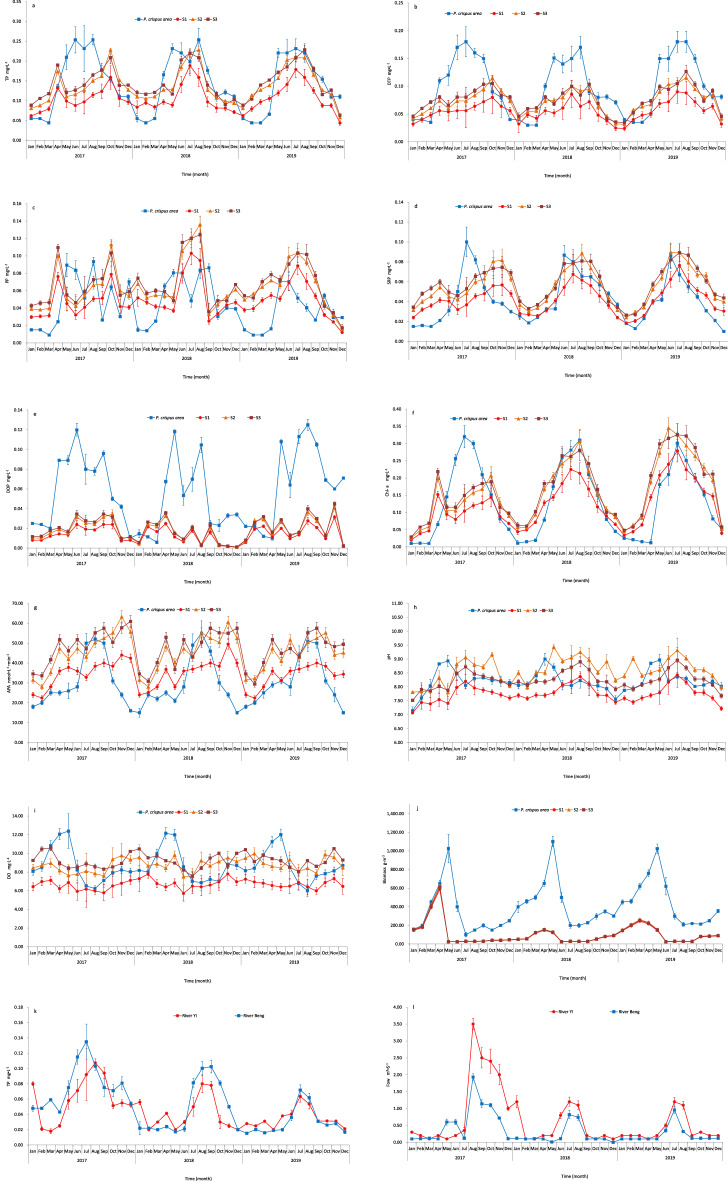
Monthly variations of water chemical and physical parameters (mean ± SD) in different areas of Yimeng Lake from January 2017 to October 2019. a-e water different P fraction concentration in different lake areas; f water chl-a concentration in different lake areas; g water APA concentration in different lake areas; h-i physical parameters in different lake areas. j biomass in different lake areas. k TP concentration of River Yi and River Beng. l the flow velocity of River Yi and River Beng.

In the two water bodies, the TP concentration was the lowest during the winter and highest during the summer (Fig. 2a). From April to June, TP rapidly increased and peaked in the summer (from May to August, TP ranged from 0.20 to 0.25 mg L^−1^). From January to March, TP remained low (0.04–0.06 mg L^−1^) in the non-harvested area for three years. In the harvested area, the TP of S1–S3 all had the same seasonal variation trend. The TP of S1 in the harvested area was significantly lower than that of the non-harvested area (*P* < 0.05). However, the TP of S2 and S3 in the harvested area were not significantly different from those in the non-harvested area (*P* > 0.05). Meanwhile, the TP of S2 and S3 remained relatively low in 2017 and showed its first peak in April. From May to October, the TP gradually increased and showed its second peak in October. From 2018 to 2019, the TP trend of the harvested area changed in the same way as the non-harvested area, but the TP concentration was relatively low in the summer and high in the winter.

In the non-harvested area, DTP exhibited roughly the same trend as TP and peaked in the summer (from May to August, the DTP ranged from 0.12 to 0.18 mg L^−1^). In the harvested area, DTP exhibited an increasing trend, with the highest concentration detected in August 2019. The DTPs of S1–S3 in the harvested area were significantly lower than those in the non-harvested area (*P* < 0.05). The DTP of S1 was lower than those of S2 and S3 from 2017 to 2019. From winter to summer, DTP gradually increased and plateaued when compared to the non-harvested area (Fig. 2b). The change trend in PP was not obvious in either water body but seemed to increase in the summer and decrease in the winter (Fig. 2c). The PPs of S1–S3 showed no significant difference between the harvested and non-harvested areas (*P* > 0.05). Data from different years showed that there was no significant difference between the harvested and non-harvested areas of PP in 2017–2018, but the content of PP in the harvested area increased significantly in 2019 (*P* < 0.05). In the non-harvested area, SRP increased rapidly from May to July in 2017 (0.03–0.10 mg·L^−1^) and increased again from May to June in 2018 and 2019. After it peaked, the SRP decreased gradually (Fig. 2d). In the harvested area, the SRP of S1 in the harvested area was significantly lower than that of the non-harvested area (*P* < 0.05). The SRP of S2 and S3 showed no significant difference between the harvested and non-harvested areas (*P* > 0.05). The SRP showed its first peak in April and its second peak in November in 2017; it also peaked in August and July in 2018 and 2019, respectively. The SRP peaks in the harvested area lagged behind those in the non-harvested area. Generally, TP, DTP, and SRP exhibited increasing trends in the harvested area but decreasing trends in the non-harvested area from 2017 to 2019.

In the non-harvested area, DOP was maintained at a high level (0.05–0.13 mg·L^−1^) and increased from May to September. It was significantly higher in the non-harvested area than in the harvested area (*P* < 0.05). There was no significant difference in DOP between S1–S3 (*P* > 0.05). DOP was maintained at a low level and changed slightly in the harvested area (Fig. 2e). TP, DTP, PP, SRP, and DOP increased in the non-harvested area and were consistent with the senescence period of *P. crispus*.

Changes in the Chl-a content exhibited the same trend as TP, DTP, and SRP. The Chl-a content had a positive relationship with the TP, DTP, and SRP concentrations in the non-harvested and harvested areas (Fig. 2f). After combining the data from the two water bodies, this relationship was still observed. In 2017, in the harvested area, the Chl-a content significantly decreased (*P* < 0.05) after the *P. crispus* was harvested. Additionally, in 2017, the content of Chl-a in the harvested area was significantly lower than that in the non-harvested area (*P* < 0.05). The Chl-a content was significantly higher in the harvested area when compared to the non-harvested area during the growth period of *P. crispus* from 2018 to 2019. On the seasonal scale, the Chl-a content was the lowest during the winter and highest during the summer. In April, the Chl-a content sharply increased when *P. crispus* began to decompose.

The total APA was maintained at a high level (46.02–55.01 nmol L^−1^ min^−1^) from July to September in the non-harvested area. In the harvested area, the total APA in S1 was significantly lower than in S2 and S3 (*P* < 0.05). APA of S1 has no significant difference with the non-harvested area(*P* > 0.05). APA of S2 and S3 was significantly higher than that of non-harvested area (*P* < 0.05). The total APA was maintained at a relatively high level from April to December. In the two water bodies, the total APA was the lowest during the winter and highest during the summer (Fig. 2g).

The pH and DO increased during the growth period of *P. crispus* from March to May and decreased during the decomposition period in the non-harvested area. In the harvested area, the pH was the lowest during the winter and highest during the summer, while the DO seemed to exhibit the opposite trend (Fig. 2h, i). The pH and DO of S1–S3 showed no significant difference between the harvested and non-harvested areas (*P* > 0.05). The pH and DO of S1 were lower than those of S2 and S2. The biomass in the harvested area decreased significantly, but as time passed, the biomass showed an upward trend (Fig. 2j).

The rainy season in the region is from July to August, and during it the amount of water in the Yi and Beng rivers increases significantly. Because the water carries a large amount of nutrients, this led to the water phosphorus content also showing a clear upward trend during the period examined (Fig. 2k, l). From 2017 to 2019, the flow of the Beng and Yi rivers decreased in the rainy season, and the phosphorus content in the water also decreased with the decrease of flow.

### Monthly variations in the sediment P fractions

**Figure 3:**
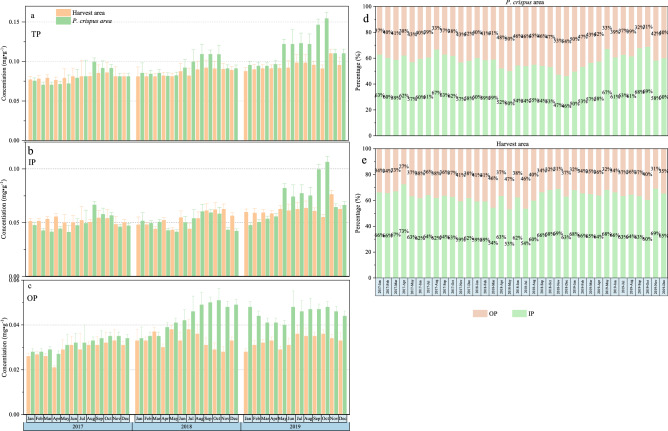
Monthly variations of sediment P fractions (mean ± SD) in different areas of Yimeng Lake from January 2017 to October 2019.**a–c** different P fractions in sediment. **d** percentage of IP and OP in the harvest area, **e** percentage of IP and OP in the *P. crispus* area.

The sediment of different P fraction concentrations exhibited increasing trends in the two water bodies. The sediment TP increased during the decomposition period of *P. crispus* from June to October in the non-harvested area. In the harvested area, the sediment TP changed slightly and exhibited an increasing trend during the summer alongside a small amount of *P. crispus* decomposition (Fig. 3a). The IP roughly exhibited the same change trend as the sediment TP in the two water bodies. The IP was higher from June to October in the non-harvested area when compared to the harvested area in 2019 (Fig. 3b). In the non-harvested area, the OP increased during the decomposition of *P. crispus*. The OP exhibited an increasing trend after 2018. Most of the time, the OP in the non-harvested area was higher than in the harvested area. In the harvested area, the OP was maintained at a relatively stable level (Fig. 3c).

The sediment P in the two areas was mainly formed by IP. The average percentages of the IP in the non-harvested and harvested areas were 58.26% and 63.51%, respectively. The OP percentage in the non-harvested area was higher than in the harvested area. The average percentages of the OP in the non-harvested and harvested areas were 41.74% and 36.49%, respectively (Fig. 3d, e). Thus, *P. crispus* harvesting clearly increased the IP proportion in the sediment.

### Comparison of the mean water and sediment indices between the harvested and non-harvested areas

No significant differences were detected in the water TP and SRP between the non-harvested and harvested areas (Table 1). The DTP and DOP were significantly lower in the harvested area when compared to the non-harvested area (*P* < 0.05). The PP exhibited the opposite trend (*P* < 0.05). The Chl-a, pH, and DO did not significantly differ between the non-harvested and harvested areas. The APA was significantly higher in the harvested area (*P* < 0.05).

**Table 1:** Tukey’s post hoc analysis of the two-way ANOVA test between Harvest area and *P. crispus* area of water and sediment chemical and physical parameters (means ± SD).

	water										Sediment		
	TP	DTP	PP	SRP	DOP	Chl a	APA	Biomass	pH	DO	TP	IP	OP
	mg L^−1^	mg L^−1^	mg L^−1^	mg L^−1^	mg L^−1^	mg L^−1^	nmol L^−1^·min^−1^	g		mg L^−1^	mg g^−1^	mg g^−1^	mg g^−1^
Harvest area	0.13 ± 0.04	0.07 ± 0.02	0.06 ± 0.02	0.05 ± 0.02	0.02 ± 0.01	0.15 ± 0.08	42.36 ± 7.96	104.26 ± 118.27	8.17 ± 0.33	8.2 ± 0.57	0.09 ± 0.01	0.06 ± 0.01	0.03 ± 0.01
*P. crispus* area	0.14 ± 0.07	0.10 ± 0.05	0.04 ± 0.03	0.04 ± 0.02	0.06 ± 0.04	0.13 ± 0.11	30.14 ± 12.35	405.72 ± 258.31	8.2 ± 0.39	8.66 ± 1.8	0.10 ± 0.02	0.06 ± 0.02	0.04 ± 0.01
F value	1.18	10.94	6.73	3.59	37.86	0.57	24.90	40.54	0.19	2.10	7.27	0.24	36.07
*P* value	0.28	0.00	0.01	0.06	0.00	0.45	0.00	0.00	0.67	0.15	0.01	0.63	0.00

In the sediment (Table 1), the TP and OP were significantly lower in the harvested area when compared to the non-harvested area (*P* < 0.05). The IP did not significantly differ between the non-harvested and harvested areas.

### Relationship between the Chl-a, environmental factors, and P composition in the water based on PCA

To study the relationship between Chl-a and water chemical and physical parameters, PCA was conducted for data reduction purposes. The indices related to Chl-a were reduced in the two areas. The two main factors (F_h_ and F_p_) were extracted in the two study areas (F_h_ in the harvested area, F_p_ in the non-harvested area). The coefficient matrix is shown in Table 2. Factor scores were calculated according to the following formulas:

1 $${\text{F}}_{{\text{h}}} = \, 0.{\text{22Z}}_{{{\text{TP}}}} + \, 0.{2}0{\text{ Z}}_{{{\text{DTP}}}} + \, 0.{\text{19Z}}_{{{\text{PP}}}} + \, 0.{2}0{\text{Z}}_{{{\text{SRP}}}} - \, 0.{\text{15Z}}_{{{\text{DO}}}} + \, 0.{\text{19Z}}_{{{\text{pH}}}}$$

2 $${\text{F}}_{{\text{p}}} = \, 0.{\text{26Z}}_{{{\text{TP}}}} + \, 0.{\text{25 Z}}_{{{\text{DTP}}}} + \, 0.{\text{21Z}}_{{{\text{PP}}}} + \, 0.{2}0{\text{Z}}_{{{\text{SRP}}}} + \, 0.{\text{22Z}}_{{{\text{DOP}}}}$$

where Z_TP_, Z_DTP_, Z_PP_, Z_SRP_, Z_DOP_, Z_DO_, and Z_pH_ are the z-standardized data of the TP, DTP, PP, SRP, DOP, DO, and pH, respectively.

**Figure 4:**
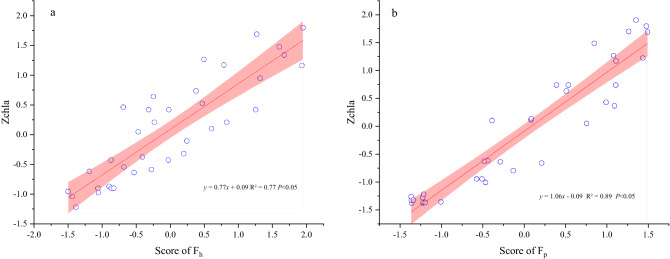
The relationship between Z score of Chl a and score of F_h_ and F_p_.

Since PCA was based on z-standardized data, the correlation analysis of the Chl-a content was also based on the z-standardized data. The Z-standardized score of the Chl-a content positively correlated with F_h_ and F_p_ (Fig. 4a, b).

**Table 2:** PCA score coefficient matrix of harvest area and *P. crispus* area.

Harvest area		*P. crispus* area	
Items	F_h_	Items	F_p_
TP	0.22	TP	0.26
DTP	0.20	DTP	0.25
PP	0.19	PP	0.21
SRP	0.20	SRP	0.20
DO	− 0.15	DOP	0.22
pH	0.19

The relationship between the Chl-a content, environmental factors, and P concentration in the water is expressed as follows:

1. Harvested area:
  3 $${\text{Z}}_{{\text{Harvested area Chla}}} = \, 0.{\text{17Z}}_{{{\text{TP}}}} + \, 0.{\text{16 Z}}_{{{\text{DTP}}}} + \, 0.{\text{14Z}}_{{{\text{PP}}}} + \, 0.{\text{16Z}}_{{{\text{SRP}}}} - \, 0.{\text{12Z}}_{{{\text{DO}}}} + \, 0.{\text{15Z}}_{{{\text{pH}}}} + \, 0.0{9}$$

(2) Non-harvested area:
  4 $${\text{Z}}_{{{\text{Non}} - {\text{harvested area Chla}}}} = \, 0.{\text{27Z}}_{{{\text{TP}}}} + \, 0.{\text{26 Z}}_{{{\text{DTP}}}} + \, 0.{\text{22Z}}_{{{\text{PP}}}} + \, 0.{\text{21Z}}_{{{\text{SRP}}}} + \, 0.{\text{23Z}}_{{{\text{DOP}}}} - \, 0.0{9}$$

Comprehensive analysis revealed that the Chl-a content in the water body was affected by the contents of different P forms in the water body and environmental factors in the harvested area. However, the Chl-a content in the water body was only affected by the content of various P forms in the water body of the non-harvested area. Clearly, the harvesting of *P. crispus* changed the influencing factors in the water body on the Chl-a content.

### Effects of harvesting on water P composition

**Figure 5:**
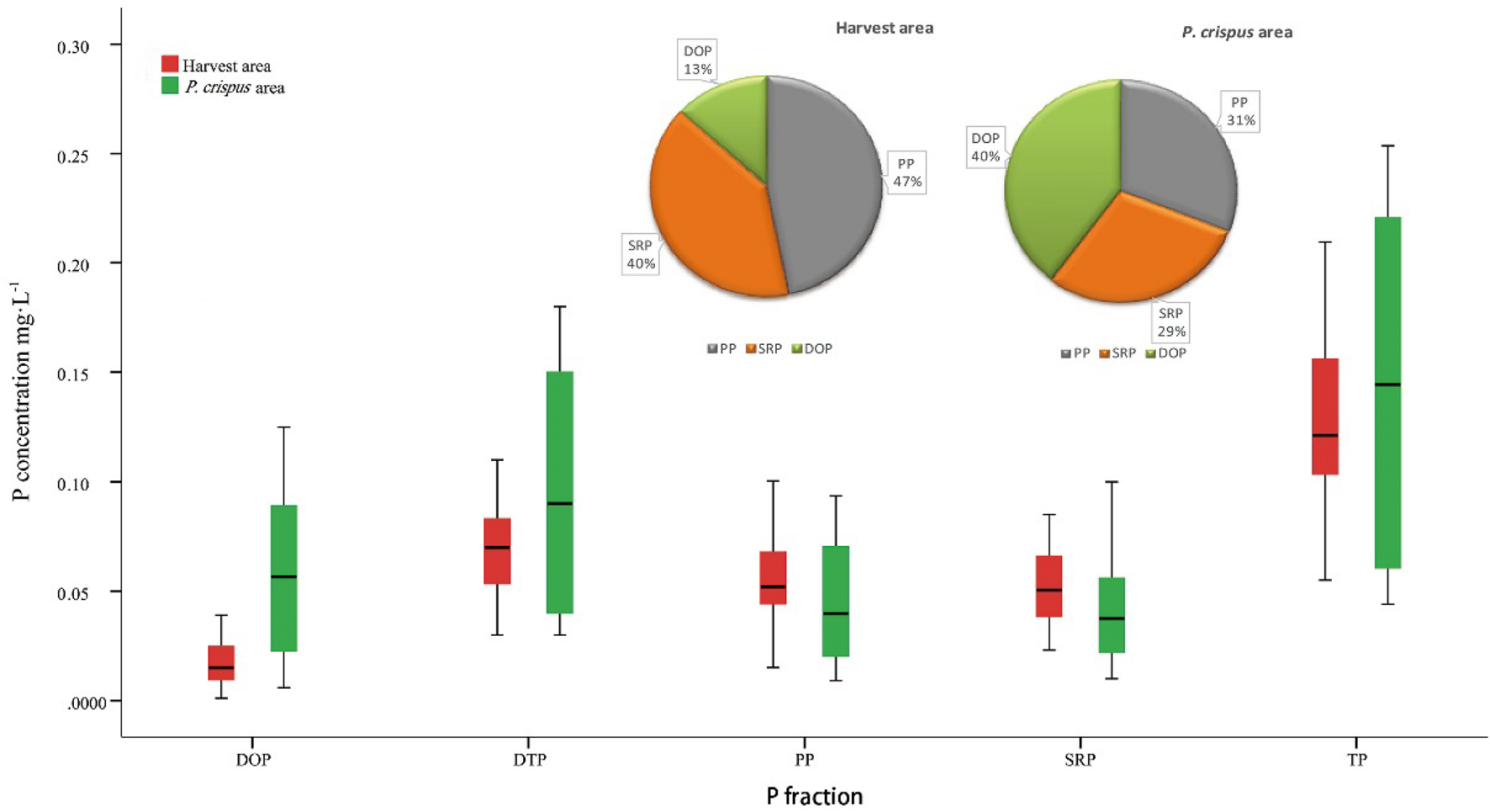
Box plots of different P fractions and P structure in water in different areas of Yimeng Lake.

Our results suggested that harvesting *P. crispus* significantly decreased the P concentration in terms of DTP, PP, and DOP in the water column; TP did not significantly decrease. Harvesting *P. crispus* increased SRP but not significantly. Fluctuations of different P fractions in the water of the non-harvested area were larger than in the harvested area. In the non-harvested area, the P in the water was mainly formed by DOP (40%), which was due to the growth and decomposition of *P. crispus* (Fig. 5), especially its decomposition.

## Discussion

### Effects of harvesting on P composition in the water

Harvesting aquatic macrophytes is an effective way to remove nutrients from water^23^. The release of phosphorus from the decomposition of submerged vegetation generally takes about 30 days under indoor simulation conditions^8^. Therefore, a suitable time to harvest the submerged vegetation must be selected to effectively prevent the pollution caused by the decomposition of submerged vegetation. In the first year after the harvesting of *P. crispus*, the content of all forms of phosphorus in the water body of the harvested area decreased significantly. The harvesting of *P. crispus* can significantly reduce the total amount of phosphorus released into the water body by the decomposition of *P. crispus* in the summer. The seasonal fluctuations in the phosphorus content in the water body in the harvested area is closely related to the seasonal fluctuations of the upstream water.

Our results are consistent with the findings of Cao, et al.^24^, who reported that *P. crispus* released a large amount of DOP after decomposition. Different P concentrations in the Beng river part of the lake, which had submerged vegetation, were significantly lower than those in the connected lake during the growth period of *P. crispus*, suggesting that presence of *P. crispus* decreased the bioavailable P in the water column^5^. The DOP concentration in the Beng river part of the lake increased and exceeded that in the Yi river part of the lake when the vegetation decayed, indicating that an amount of OP was released in the decomposition process of *P. crispus* in the Beng river part of the lake^6^. During the decomposition period of *P. crispus* from May to August, the content of DOP in the water body of the Beng river increased significantly—the average DOP content during this period was 0.89 ± 0.03 mg L^−1^—while the average DOP content in the water body of the Yi river was only 0.03 ± 0.01 mg L^−1^ in the same period. The submerged macrophyte P. crispus has consistently been found to increase the concentrations of DOP during the decomposition period in the lake^8^. During summer and autumn, the decomposition of the submerged macrophyte *P. crispus* is a major source of biologically available P and decomposable dissolved organic matter for the phytoplankton community of the lake, suggesting that *P. crispus* decomposition potentially stimulates phytoplankton production^25^. Contrary to our results, Stephen, et al.^26^ hypothesized that the submerged macrophyte *P. crispus* increased the sediment release rate of P during the growing season. *P. crispus* absorbed phosphorus in sediment during its growth period and released phosphorus into the water during its decomposition period. *P. crispus* growth resulted in increased P concentrations in the overlying waters and transported P from the sediment to the overlying water^5^. In short, the decomposition of *P. crispus* increased the turnover rate of DOP in our research results, indicating a higher bioavailability of phosphorus from DOP, and that the senescence of *P. crispus* changed the quality and quantity of the P supply in the water.

In the harvested area, PP was the main component of P in the water (47%). The water body was greatly disturbed by wind and waves in the absence of *P. crispus*, thereby increasing the PP content (Fig. 5). The percentage of the SRP was 40% in the absence of *P. crispus*. Changes in environmental regulations lead to desorption, reduction, or dissociation reactions of unstably bound P on the surface of some particles, which are converted to SRP and other P fractions^27^. Therefore, high percentages of the SRP and PP increase the risk of eutrophication.

Compared with the non-harvested area, the percentage of DOP in the harvested area showed a downward trend, while the percentage of PP and SRP showed an upward trend (Fig. 5). Therefore, it can be inferred that harvesting *P. crispus* can change the dominant form of phosphorus in the water from DOP to PP and SRP.

TP decreased in summer in the harvested area. However, different P fractions increased in the non-harvested area at the same time, indicating that *P. crispus* harvesting reduced the P concentration in the water. The TP and other P fractions increased and reached their second peak in October 2017, which may be due to the decomposition of algae and residual aquatic plants. The release of P in the sediment increases the P in the water body. Changes in environmental factors lead to the release of P in the sediment. The pH and DO significantly decreased after *P. crispus* harvesting; a low pH and DO also leads to P release. The data from 2018 and 2019 showed that the P concentration in the water recovered the increasing trend during the summer and decreasing trend during the winter after one year of harvesting. Collectively, these findings show that *P. crispus* harvesting can reduce the P concentration within a short period of time.

P is a key element that limits the primary productivity in the water body, as evidenced by the significant positive relationship detected between the P concentration and Chl-a in many freshwater lakes^28^. The mean SRP concentration in the non-harvested area was significantly lower than in the harvested area, suggesting that the presence of *P. crispus* decreased the bioavailable P in the water column. The decomposition of *P. crispus* is a major source of bioavailable P and decomposable dissolved organism matter (DOM) for the phytoplankton community in a lake, suggesting that macrophyte decomposition potentially stimulates phytoplankton production^29^. The decomposition of *P. crispus* increased the turnover rate of the DOP, indicating the presence of more bioavailable DOP mediated by alkaline phosphatase^24^.

### Effects of harvesting on environmental factors in the water

Alkaline phosphatase is a type of phosphate hydrolase with wide specificity that can catalyze the hydrolysis reactions of all phosphate esters and transfer reactions of phosphate groups. It directly participates in P metabolism, provides P for the rapid growth of plankton in the water body, and plays an important role in the transformation of P in aquatic ecosystems^30^. APA is increased during the summer and decreased during the winter in the two areas of this study. The ability of phytoplankton to excrete extracellular phosphatase that catalyzes DOP to overcome P deficiency is a possible explanatory reason^31^. For many lakes, most of the TP in the overlying water is in the form of OP, in which, > 70% is particulate OP in the suspension, while the rest is dissolved OP in colloidal form. However, the dissolved inorganic orthophosphate content that directly enters the cell body and is absorbed and utilized by phytoplankton only accounts for 5%, which changes quickly due to its direct utilization^32^. In the absence of orthophosphate in the water, alkaline phosphate can be induced in algae and bacteria as an inducer for hydrolyzing monophosphate. Submerged macrophytes can reduce the maximum reaction rate of alkaline phosphatase in overlying water^33^. Kalinowska^34^ found that in lakes dominated by submerged macrophytes, the decomposition of OP by phosphatase was almost negligible. The reason may be that more enzymes were inactivated due to the complexation of humus. *P. crispus* changed the composition of alkaline phosphatase in the water, thereby reducing the maximum enzymatic reaction rate. A previous study demonstrated that the total APA in an Indian pond system was proportional to the TP and total bacteria in the water^35^. Additionally, the resuspension of sediment led to a higher P content in the particles, which promoted the reaction rate of alkaline phosphate. Zhou and Dodds^36^ showed that the APAs associated with different-sized particles had different kinetic characteristics. A similar phenomenon was observed in Lake Donghu and Lake Nanhu in China^37^. In this study, the APAs of S2 and S3 was significantly higher than those of the non-harvested area. This means that harvesting *P. crispus* led to the increase of water APA.

Phytoplankton are important producers in river ecosystems and play important roles in the material circulation and energy transformation processes^38^. Chl-a is a comprehensive performance that reflects physical and chemical factors and biological indicators in the water body; it is also restricted by environmental factors^39^. Therefore, Chl-a can be used to characterize the growth state of phytoplankton in the water.

The Chl-a content had significant positive relationships with the TP, DTP, PP, SRP, and pH, but a negative relationship with the DO in the harvested area, while in the non-harvested area, it had significant positive relationships with the TP, DTP, PP, DOP, and SRP (Table 3). The Chl-a content in the non-harvested area was mainly affected by the P content of the water body, while in the harvested area, Chl-a was affected by the P content and environmental factors in the water body.

**Table 3:**
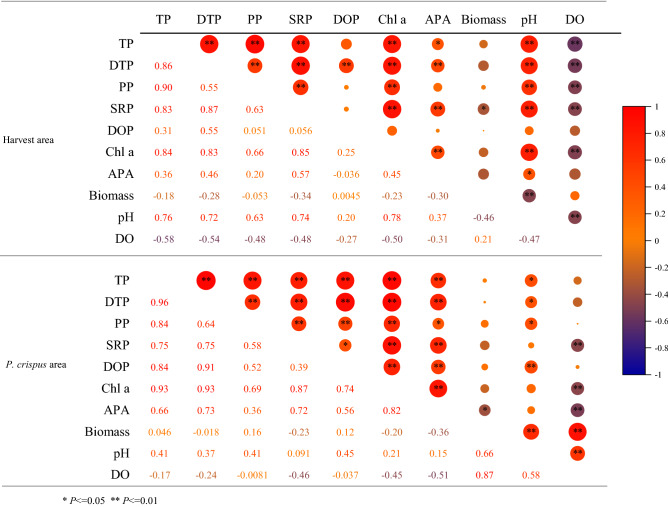
Pearson's correlation coefficients describing the relationships between water chemical and physical parameters in the two studied areas.

There is a close relationship between APA content and Chl-a content. The correlation coefficient between APA and Chl-a in the non-harvested area with *P. crispus* is 0.82, while the correlation coefficient between APA and Chl-a in the harvested area is 0.45. This indicates that the harvest of *P. crispus* decreased the correlation between APA and Chl-a.

The Chl-a content in the harvested area decreased sharply in the first year of harvesting, but as time passed, the Chl-a in the water returned to a higher level in the second and third years. Therefore, harvesting can only improve the water quality for a short time; examining water quality in the long term revealed that *P. crispus* harvesting had no clear sustained effect on improving water quality.

Through PCA, F_h_ and F_P_ can be used to represent the physical and chemical indexes of the harvested area and the non-harvested area with *P. crispus*, respectively. Linear regression analysis of the F_h_, F_P_, and Z_chl-a_ scores showed that harvesting *P. crispus* could reduce the slope of the linear equation between the principal component score and Z_chl-a_ (Fig. 4). Therefore, the chlorophyll content in the non-harvested *P. crispus* area is more sensitive to changes in physical and chemical environmental indicators.

### Effects of harvesting on sediment P fractions

The migration and transformation of P by *P. crispus* remains controversial. One notion is that *P. crispus* activates the P in the sediment, wherein *P. crispus* mainly absorbs the P in the sediment during its growth period, then releases it into the water body during its decomposition period^40^. Therefore, *P. crispus* serves a P pump role and activates the P in the sediment into the water, which intensifies eutrophication. Another explanation is that *P. crispus* metabolizes easily-released P into stable P. *P. crispus* releases P into the water body during the decomposition period for a relatively short period of time^8,41^. After a month, the P in the water body returns to a relatively low level. The growth cycle of *P. crispus* absorbs easily released forms of P in the sediment and then converts them into difficult-to-release forms of P, which are finally deposited in the sediment for permanent storage. *P. crispus* can also cause P adsorption in the sediment^15^. This increased P capacity indicates that the final migration direction of P is to the sediment.

In this study, the TP, IP, and OP contents in the sediment of the non-harvested area exhibited upward trends, indicating that the migration direction of P in the water body was to the sediment after a long adjustment period by *P. crispus*. The OP percentage in the non-harvested area was higher than in the harvested area, indicating that under the action of *P. crispus*, P forms in the sediment migrate in the direction of OP.

*P. crispus* can absorb phosphorus in water body to the plant itself during the growth period. Therefore, harvesting *P. crispus* can effectively reduce the total amount of phosphorus in the water body. However, this study showed that harvesting *P. crispus* destroys the original water environment. Over the long term, the chlorophyll content in the harvested water body increases over time, which may lead to a cyanobacteria bloom in the water body.

Endogenous P in the sediment is mainly derived from the precipitation of PP in lakes but dissolved P (after adsorption by mineral particles) and OP in organisms. The migration and transformation process of P at the sediment–water interface is a key process of the P cycle in eutrophic lakes and is affected by many factors, including sediment properties, the interface environment, and biological characteristics^5^. The classical P cycle theory suggests that P will be adsorbed by iron (hydrogen) oxides or precipitate FePO_4_ and that the release of P is mainly controlled by DO and the redox potential^27^. In recent years, scholars have conducted several studies on the migration and transformation mechanism of P at the sediment–water interface and gained a certain understanding of the influence mechanism of environmental and biological factors^42–44^. For example, DO will affect the decomposition rate of organic matter and occurrence of P, pH will affect the combination of P and metals, such as Fe and Al, and temperature will affect the activity of microorganisms and decomposition rate of organic matter, thereby affecting the migration and transformation of P.

In this study, *P. crispus* released a large amount of organic matter into the water and sediment during the decomposition process, thereby changing environmental factors in the water body. The pH and DO increased during the growth period of *P. crispus* and decreased during the decomposition period in the non-harvested area. The increase in the TP content in the sediment may be related to the input of external P from the water body. *P. crispus* fixes external P and migrates into the sediment during the growth and decomposition processes. The increased P content in the sediment in the non-harvested area may be the result of the combined effects of these environmental factors.

The TP content in the water body of the harvested area showed an upward trend over time. The main reason for this may be that harvesting changes the environment of the water body, thus changing the phosphorus balance between the sediment and the water interface, resulting in the release of phosphorus from the sediment to the water body. As indicated by the TP content in the upstream water, the TP content in the upstream water of the Yi and Beng rivers decreased with time. Therefore, endogenous phosphorus pollution is the main reason for the increase of phosphorus content in the water body of the harvested area.

## Conclusion

During the three years of field observations, we found that *P. crispus* harvesting significantly reduced DTP and DOP and increased APA and PP in the water. No significant differences were detected in the water TP, SRP, Chl-a, pH, or DO between the non-harvested and harvested areas. The sediment TP and OP significantly decreased in the harvested area. No significant difference was detected in the IP between the non-harvested and harvested areas.

The sediment P in the two areas was mainly formed by IP. The average percentages of IP in the non-harvested and harvested areas were 58.26% and 63.51%, respectively. The OP percentage in the non-harvested area was higher than in the harvested area.

Different forms of P decreased during the growing season, while all P forms increased during the decomposition period of *P. crispus*. The growth and decomposition of *P. crispus* changed the composition of P in the water body. In the non-harvested area, P in the water was mainly formed by DOP (40%), while PP was the main component in the harvested area (47%).

The harvesting of *P. crispus* changed the factors in the water body affecting the Chl-a content. The Chl-a content in the water was affected by different forms of P and environmental factors in the harvested area.

## Data Availability

The datasets used during the current study are available from the corresponding author upon reasonable request.
